# Integration of daytime radiative cooling and solar heating

**DOI:** 10.1016/j.isci.2022.105894

**Published:** 2022-12-27

**Authors:** Xiuqiang Li, Sujin Shao, Meijiao Huang, Shuyuan Zhang, Wanlin Guo

**Affiliations:** 1 Key Laboratory for Intelligent Nano Materials and Devices of Ministry of Education, and Institute for Frontier Science, Nanjing University of Aeronautics and Astronautics, Nanjing 210016, China

**Keywords:** Energy engineering, Energy application, Energy materials

## Abstract

In recent years, sustainable energy development has become a major theme of research. The combination of solar heating and daytime radiative cooling has the potential to build a competitive strategy to alleviate current environmental and energy problems. Several studies on the combination of daytime radiative cooling and solar heating have been reported to improve energy utilization efficiency. However, most integrations still have a low solar/mid-infrared spectrum regulation range, low heating/cooling performance, and poor stability. To promote this technology further for real-world applications, herein we summarize the latest progress, technical features, bottlenecks, and future opportunities for the current integration of daytime radiative cooling and solar heating through the switch mode (including electrical, thermal-responsive, and mechanical regulations) and collaborative mode.

## Graphical abstract

**Figure:**
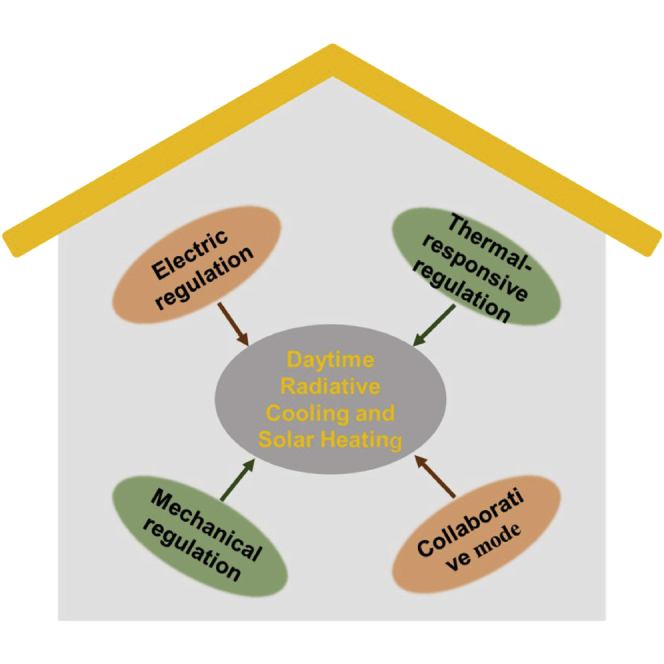

## Introduction

Buildings consume around 30% of the global energy and contribute to about 20% of global greenhouse gas emissions. Approximately 50% of the energy is consumed during space heating and cooling.^1^^,^^2^^,^^3^^,^^4^^,^^5^ Greenhouse gas emissions are the major challenge of the current energy model, which significantly impacts the climate and environment. Among the sustainable energy sources, solar heating^6^^,^^7^ and daytime radiative cooling^8^^,^^9^ are expected to provide competitive strategies to resolve these problems. Solar heating, which is the absorption of sunlight and the generation of heat through the thermal vibration of molecules, is widely applied in real-world situations. The ideal spectrum for solar heating is the one with high solar absorptivity (α) (0.2–2 μm) and low emissivity (ϵ) in the radiative spectrum (∼2–20 μm). Nighttime radiative cooling can only work at night because the cooling materials cannot adequately reflect solar energy. In 2014, Raman et al.^8^ first demonstrated the daytime radiative cooling concept by designing an integrated photonic solar reflector (with >97% reflectance) and a thermal emitter (exhibiting high emission in the atmospheric transparency window) consisting of seven alternating HfO_2_/SiO_2_ layers. The principle is that materials can effectively reflect sunlight and achieve cooling by radiating energy into deep space through the atmospheric window (8–13 μm). Ambient conditions and the optical and thermal properties of materials significantly affect the device performance. The maximum radiative cooling power was ∼120 W/m^2^.^10^

Most buildings are located in a dynamic environment (including climate zone dependence and diurnal, seasonal, and energy price fluctuations), which can compromise the efficacy of single-mode devices (heating or cooling only). The combination of solar heating and daytime radiative cooling, which can switch between heating and cooling (a dual-mode device), is conducive to improving the utilization efficiency of devices in a dynamic environment. Li et al.^11^ calculated that if the dual-mode device in the United States were widely deployed, it could have saved 19.2% (236 GJ) of heating and cooling energy (annual energy consumption for building heating and cooling is 548 and 681 GJ, respectively), which is 1.7 times higher than the cooling-only (138 GJ) and 2.2 times higher than the heating-only (106 GJ) approaches. Liu et al.^12^ calculated the energy saving of single-storage residential building. The results show that 42.4% (963.5 kWh) of electricity can be saved in the cooling season with the dual mode, and 63.7% (1449.1 kWh) of electricity can be saved when coupled with the energy storage system. For heating, 14.7% (492 kWh) of electricity can be saved. However, combining these two technologies introduces new problems, such as a low spectral regulation range, low heating/cooling performance, and poor stability.

Although this field has been widely explored in the past few years, it is still in its early stages. To further promote research in this field and effectively solve its problems, we summarize the previous results from two aspects, switch mode (including electrical,^13^^,^^14^^,^^15^ thermal-responsive,^16^^,^^17^^,^^18^^,^^19^^,^^20^ and mechanical regulations^21^^,^^22^^,^^23^^,^^24^^,^^25^^,^^26^^,^^27^^,^^28^) and collaborative mode,^29^^,^^30^ and introduce their principles, characteristics, and progress. In addition, unresolved scientific and technical issues associated with the outlook are presented in this paper. It is worth noting that we mainly focus on the combination of daytime radiative cooling and solar heating. Conversely, a combination of nighttime radiative cooling and solar heating can be found in Refs.^31^^,^^32^^,^^33^^,^^34^^,^^35^

## Switch mode

### Electric regulation

Many reports have been published on the use of electricity to tune the solar spectrum for smart windows^36^^,^^37^^,^^38^^,^^39^^,^^40^ or the mid-infrared (IR) spectrum for IR stealth/camouflage.^41^^,^^42^^,^^43^^,^^44^^,^^45^ The principle of electrical regulation is usually to regulate the redox reaction of the electrochromic material through the insertion and extraction of H^+^, Li^+^, and other ions to change the light absorption or to regulate the reflection and absorption of light through the electrochemically reversible deposition of metal. After years of development, such devices generally exhibit good regulation and stability. For example, Cai et al.^46^ prepared porous WO_3_ as an active electrochromic material using pulsed electrochemical deposition at a low cost and large scale. The porous structure can facilitate charge transfer and electrolyte penetration and alleviate the expansion and contraction of WO_3_ during H^+^ insertion and extraction. Consequently, the device exhibited excellent stability and achieved 97.7% optical modulation at 633 nm. Li et al.^45^ tuned IR emissivity by reversibly depositing Ag onto nanoscopic platinum (Pt)/BaF_2_ glass (mid-IR transparent glass). Because nanoscopic Pt has strong mid-IR absorption and the electrolyte also absorbs partial IR transmission, the device exhibits high mid-IR emissivity when Ag is not deposited. Conversely, when Ag is deposited, its dense layer is formed on the surface of BaF_2_, which can strongly reflect IR and exhibit a lower emissivity. Moreover, because of the high chemical inertness of Pt and Ag, they can be deposited and dissolved multiple times, resulting in ∼0.77 mid-IR modulation range and ≥350 cycle performance.

**Figure 1:**
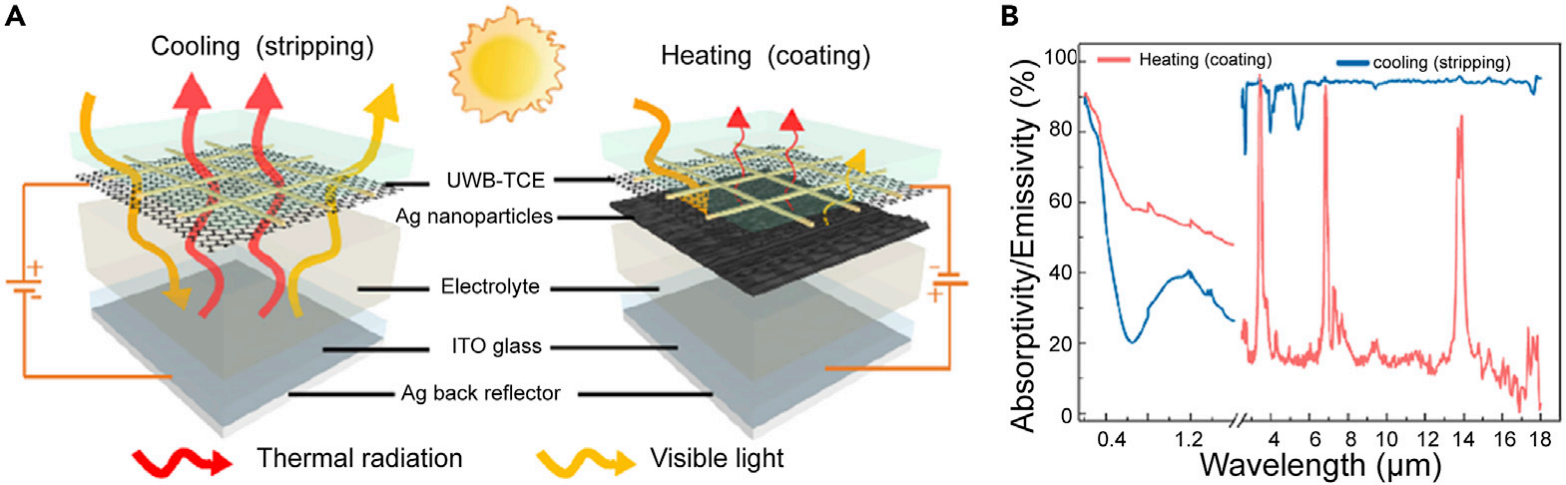
Electric regulation (A) Schematic diagram of the working principle of the electrochromic device. (B) Solar absorption spectra and infrared emissivity of devices in cooled and heated states. Reproduced with permission.^13^ Copyright 2021, American Chemical Society.

The ideal spectral range for solar heating is the one with a high α and low ϵ. Conversely, for daytime radiative cooling, the solar spectrum should have high solar reflectance and ϵ in the range 8–13 μm. Efficient heat management requires coordinated multispectral regulation (mainly visible, near-IR, and mid-IR). However, synergistic multispectral regulation based on electric regulation is still challenging because of the difficulty in obtaining long-spectral (from visible to mid-IR) transparent conductive electrodes and the unclear relationship between the deposition structure and performance.^13^^,^^14^^,^^15^ Rao et al.^13^ prepared metal meshes with low-sheet-resistance (R_s_ = 22.4 Ω/sq), high-optical-transmittance (T_UV−vis_ = 85.63%, T_near-IR_ = 87.85%, and T_mid-IR_ = 84.87%), and long-wavelength transparent conductive electrodes. Thus, the regulation of solar heating and daytime radiative cooling can be achieved. Specifically, when metal particles with appropriate sizes and distributions are deposited on transparent electrodes, broadband localized surface plasmon resonance with high solar absorption is generated. In addition, based on the effective medium theory, metallic components with high electrical conductivity usually have low mid-IR emissivity (Figure 1A, right). In this case, the device can achieve heating. When deposited in the opposite direction (cooling state), a silver film is formed on indium tin oxide glass to reflect sunlight. The electrolyte is rich in functional groups and has a high emissivity in the mid-IR region (Figure 1A, left). As a result, the spectral properties could be tuned from the heating state (α, ϵ) = (0.33, 0.94) to the cooling state (α, ϵ) = (0.60, 0.20) (Figure 1B). Although this synergistic regulation has been demonstrated, the performance of the current device still needs improvement.

### Thermal-responsive regulation

**Figure 2:**
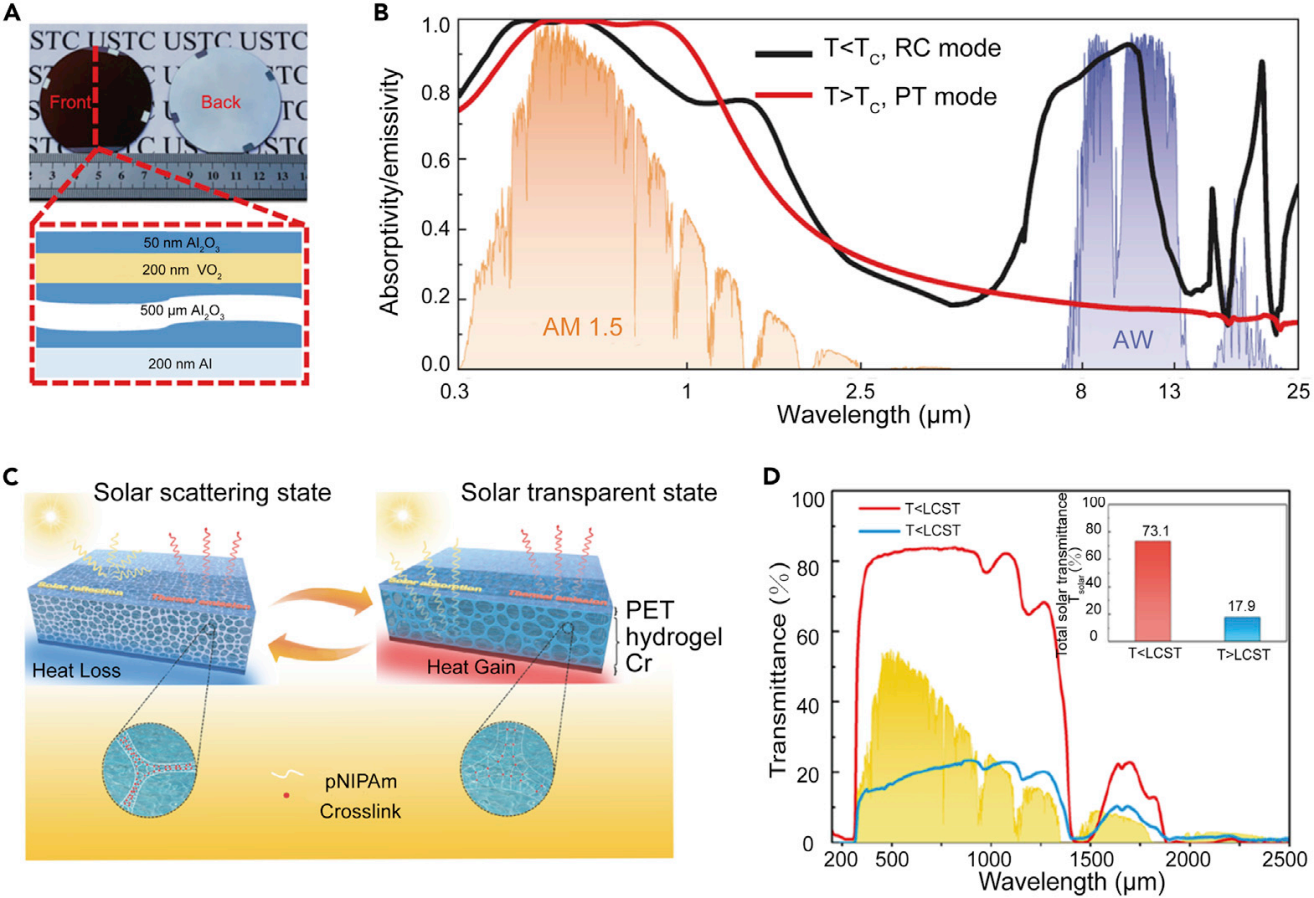
Thermal-responsive regulation (A) Photographs of the proposed spectrally self-adaptive absorber/emitter (SSA/E) with multilayer structure. (B) Simulated absorbance/emissivity of SSA/E from 0.3 to 25 μm when the temperature of SSA/E is below and above Tc. Reproduced with permission.^16^ Copyright 2022, National Academy of Science. (C) Working principle of device. The top part is the radiation cooling part (polyethylene terephthalate), the middle part is the thermochromic part (hydrogel), and the bottom part is the solar heating part (Cr). When its temperature is lower than LCST, solar radiation will reach the bottom layer through the top and middle layers and be absorbed, producing a heat gain effect; when its temperature is higher than LCST, solar radiation will be strongly scattered by the middle layer, producing a heat loss effect. (D) Solar transmittance of pNIPAm hydrogels before and after phase transformation. Reproduced with permission.^18^ Copyright 2021, American Chemical Society.

Thermal responsiveness differs from electrical regulation in that it does not require electricity and has a more straightforward construction. Currently, thermal responsiveness is mainly based on two classes of materials: VO_2_^16^^,^^17^ and poly(N-isopropyl acrylamide) (pNIPAm) hydrogels.^18^^,^^19^^,^^20^ The regulation of VO_2_ is primarily achieved via a metal-insulator phase transition. VO_2_ exhibits distinct features toward mid-IR light in these two states. The insulating state is a low-loss dielectric with high mid-IR absorption. Conversely, the metallic phase is a plasmonic metal with a high damping constant and strong mid-IR reflection. However, the phase-transition temperature of VO_2_ is high (∼68 °C). Although doping can reduce the phase-transition temperature to the required range, the regulatory performance is simultaneously reduced.^46^^,^^47^^,^^48^^,^^49^^,^^50^ In addition, VO_2_ exhibits lower regulation of the solar spectrum. Ao et al.^16^ designed a structure composed of Al_2_O_3_ (50 nm)/VO_2_ (200 nm)/Al_2_O_3_ (500 μm)/Al (200 nm), which has strong solar light absorption owing to the intrinsic light absorptivity of VO_2_ and an anti-reflection structure (Figure 2A). When the temperature is changed from 20°C (below the critical temperature [Tc]) to 80 °C (above Tc), the solar and IR spectra change from (α, ϵ) = (0.83, 0.75) to (α, ϵ) = (0.89, 0.25) (Figure 2B). The results show that in the heating state, the material can reach a temperature that is ∼170°C above ambient in vacuum under sunny conditions. A temperature drop of 20°C can be achieved in the cooling state.

pNIPAm is commonly used to modulate the spectra of hydrogels. pNIPAm possesses hydrophilic amide and hydrophobic isopropyl groups in its monomer structure, which determine its lower critical solution temperature (LCST, ∼32°C). One explanation for the regulation of solar light by pNIPAm is that when the ambient temperature is lower than the LCST, pNIPAm can adsorb water and form a hydration shell on its surface. It becomes transparent because its refractive index is similar to that of water. When the temperature exceeds the LCST, the adsorbed water is lost and the pNIPAm network shrinks. The phase separation interface between the pNIPAm network and water strongly scatters sunlight because of the refractive index contrast.^18^ As shown in Figure 2C, Fang et al.^18^ designed a three-layer structure: polyethylene terephthalate at the top as the radiative cooling part, pNIPAm hydrogel in the middle as the thermochromic part, and black chrome at the bottom as the solar heating part. When the temperature is lower than the LCST, the hydrogel is transparent and sunlight can be absorbed and utilized by the bottom black chrome. However, when the temperature is higher than the LCST, the hydrogel is in a reflective state (concurrently, the hydrogel has strong IR emissivity), which can effectively achieve cooling. After optimization, the device can tune the solar transmittance (T_solar_) from 73.1% to 17.9% when the temperature changes from below the LCST to above it (Figure 2D). Mei et al.^19^ designed a PVDF@PNIPAm film that can achieve high visible-light reflectance/transmittance modulation (ΔR_vis_ = 70.0% and ΔT_vis_ = 86.3%) and high mid-IR emissivity (0.96). The outdoor test results show that the material can have a temperature drop of 1.8°C–3.7°C during a hot day and an increase of 4.3°C–5.8 °C during a cold day. The thermal responsiveness of the hydrogel-based system is still not very efficient because the mid-IR spectrum is always in a high-emission state during the tuning process, which depletes the power of solar heating.

### Mechanical regulation

Solar heating and daytime radiative cooling materials can usually be designed separately for mechanical regulation, such as side-by-side or Janus that is switched by mechanical movement. Owing to this principle, the current development of mechanical regulation is mature in terms of performance and stability compared with the above-mentioned two methods. Thus far, several methods for achieving mechanical regulation have been developed, such as roll-to-roll,^12^ Janus,^21^^,^^22^^,^^23^^,^^24^ switchable,^25^^,^^26^^,^^27^ and programmable.^28^ Zhao et al.^25^ designed a bilayer film (porous silicone/carbon black particles) that can be switched between a transparent solid state for solar heating and a highly porous state for daytime radiative cooling (solar reflection and radiative cooling) through compression and stretching processes. Specifically, it can achieve 95% solar absorption in the transparent state, increasing the ambient temperature from 10°C to 28°C in cold weather. Moreover, the porous state of the bilayer film can achieve 93% solar light reflection and 94% mid-IR emissivity, resulting in a 5°C temperature drop in hot weather (∼35 °C). Even though the material exhibits excellent performance, it is always in a high-emission state during compression and stretching.

**Figure 3:**
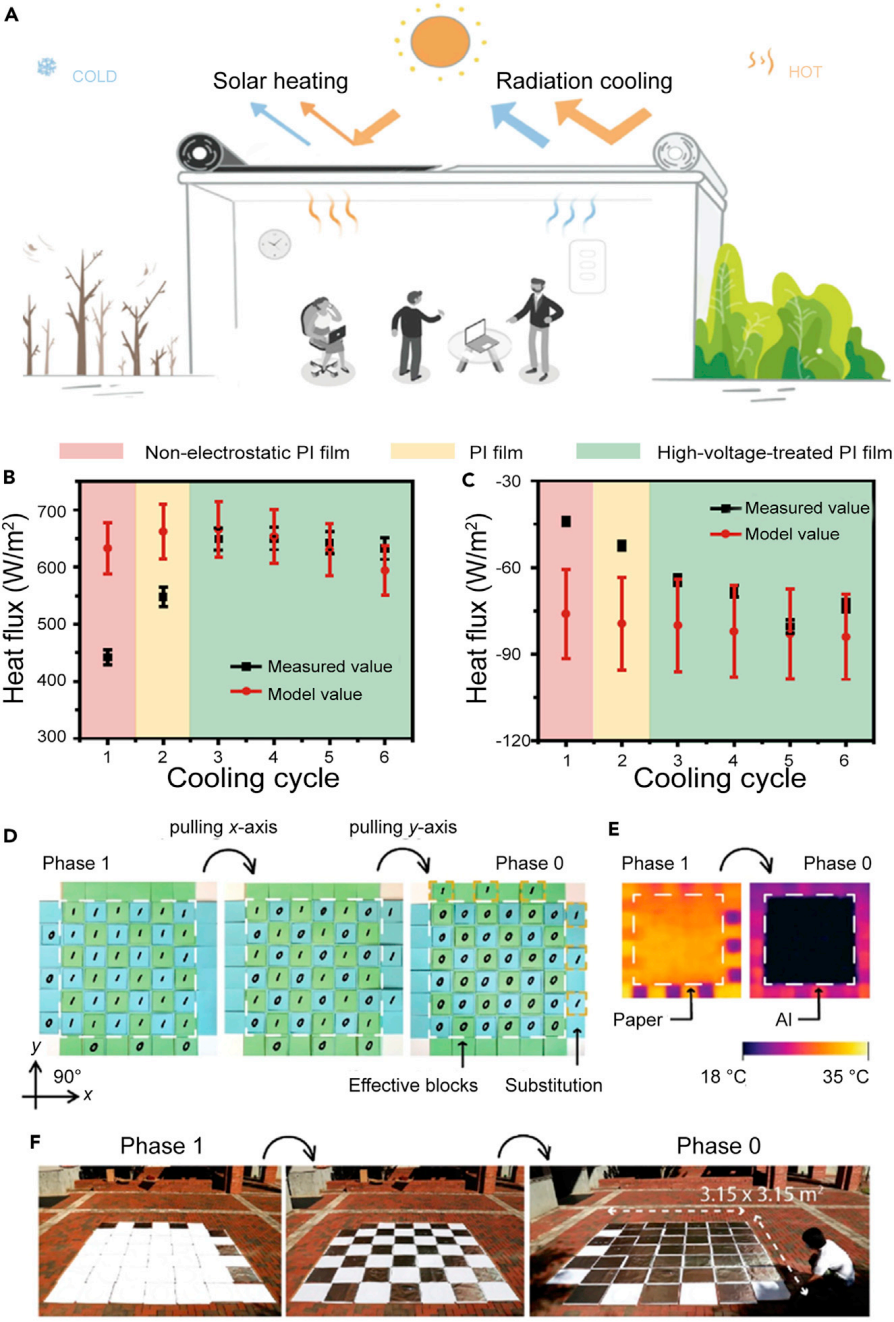
Mechanical regulation (A) Schematic diagram of the dual-mode device in heating (left) and cooling (right) modes. (B) Average solar heating flux over different cycles. (C) Average radiative cooling heat flux over different cycles. The first cycle was performed using a non-electrostatic polyimide (PI) film (red area), the second cycle used PI film (yellow area), and the rest of the cycles used PI film with external voltage treatments (green area). Reproduced with permission.^11^ Copyright 2020, Springer Nature. (D) Photographs of the complete transition from phase 0–1 by pulling the x- (blue paper) and y- (green paper) bands. (E) IR image showing the complete transition from the paper-based high-ϵ_LWIR_ to the aluminum-based low-ϵ_LWIR_ surface. The samples were heated on a ∼30°C hot plate. (F) Demonstration of the surface transition in a 3.15 × 3.15-m^2^ aluminum paper sample. Reproduced with permission.^28^ Copyright 2022, American Chemical Society.

To achieve multi-spectrum coordinated regulation, Li et al.^11^ designed high-performance solar heating (Cu nanoparticles/Zn film, 93.4% absorption in 300–2000 nm and 14.2% emissivity in mid-IR) and radiative cooling materials (Polydimethylsiloxane [PDMS]/Ag, 97.3% reflectance in 300–2000 nm and 94.1% emissivity in 8–13 μm). The research group achieved switching between solar heating and daytime radiative cooling using the roll-to-roll method (Figure 3A). Benefiting from the solution of the contact thermal resistance between the film and the substrate by applying static electricity, the results show that the device can achieve up to 71.6 W/m^2^ of cooling power density and 643.4 W/m^2^ of heating power density (Figures 3B and 3C). Ke et al.^28^ demonstrated a programmable interwoven surface that can dynamically switch the overlapping sequence to regulate solar heating and daytime radiative cooling (Figures 3D and 3E). Moreover, the size of the device can reach 3.15 m (Figure 3F). The experimental results show that the device can achieve 0.87 ± 0.01 solar light absorption and 0.38 ± 0.03 mid-IR emission in the heating state. In the cooling state, the solar light absorption is 0.05 ± 0.01, and the emissivity of the mid-IR is 0.91 ± 0.03. Thus, mechanical regulation achieves significant progress in the design of performance and size. However, the understanding of the boundary conditions for the application of such mechanical regulation requires further studies.

## Collaborative mode

**Figure 4:**
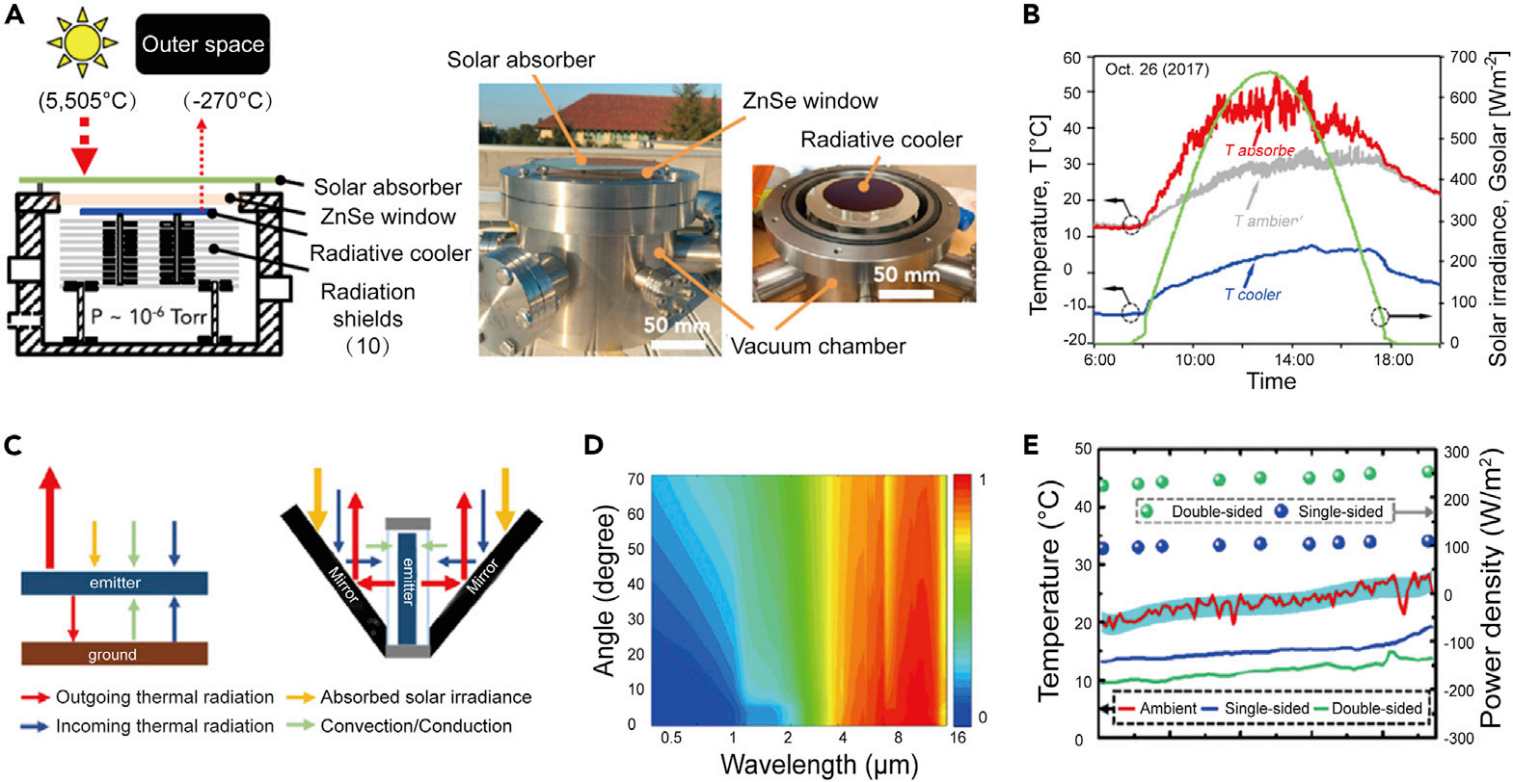
Collaborative mode (A) Schematic diagram of the experimental setup (left). The solar absorber is an undoped germanium wafer with a double-sided anti-reflective coating with high absorption in the solar spectrum and high transparency in 8–13 μm. The ZnSe window is used to achieve high vacuum mechanically and is also infrared transparent. *In situ* experimental setup (right). (B) Temperatures of T_absorber_ (red), T_ambient_ (gray), and T_cooler_ (blue) over time in the outdoor testing. The difference between the temperatures of the T_cooler_ (blue) and T_ambient_ is fairly constant, reaching a maximum value of 28.9°C at 16:30, indicating significant radiative cooling in the sunlight. Reproduced with permission.^29^ Copyright 2018, Cell Press. (C) Power trade-off diagram for a conventional single-sided radiant cooling system (left panel) and a double-sided system (right panel). (D) Measured angle-dependent reflectance of the selective solar absorber. (E) Best cooling performance was measured in Buffalo, NY, on May 21, 2019. Solid curves indicate real-time ambient temperatures. Spheres indicate the extracted cooling power density for both bilateral and unilateral systems. Reproduced with permission.^30^ Copyright 2021, Cell Press.

Building’s needs for heating and cooling are sometimes simultaneous.^11^ From the perspective of efficient energy utilization, it is ideal for solar heating and radiative cooling to work independently and simultaneously in the same physical area (the same is true for gable roofs). However, solar heating and daytime radiative cooling are mutually exclusive. On the one hand, the solar heating device will act as a heat source to transfer heat to the cooling device, weakening its cooling power. However, the spectral utilization of daytime radiative cooling and solar heating was reversed. To resolve this issue, Chen et al.^29^ first proposed and achieved a collaborative mode that can simultaneously realize the independent work of solar heating and radiative cooling. Specifically, as shown in Figure 4A, they chose a transparent mid-IR solar absorber (500-mm-thick undoped germanium) and placed it above the radiative cooler (>80% emissivity at 8–13 μm). The vacuum chamber blocked the heating effect of the solar absorber and the environment on the radiative cooler. The experimental results show that solar heating can achieve a temperature 24.4 °C higher than the ambient temperature, and the radiative cooler can reach a low temperature of 28.9°C below room temperature (Figure 4B). The performance of the system can be further improved by using an efficient mid-IR transparent absorber. Furthermore, Zhou et al.^30^ achieved simultaneous solar heating and radiative cooling using two selective absorbers. As shown in Figure 4C, the selective absorber absorbs sunlight (α > 92%, >90% reflectance in mid-IR, Figure 4D) and acts as a mirror to redirect the thermal emission from a vertically radiative cooler. Owing to this specific structure, both sides of the radiative cooler can be used together to achieve a cooling power density of over 270 W/m^2^, a temperature drop of 14°C in the laboratory environment, and a temperature drop of over 12°C in outdoor testing (Figure 4E).

## Outlooks

With the advancement of daytime radiative cooling, many studies combining solar heating and daytime radiative cooling have been reported in recent years. We systematically summarize the background, progress, and challenges of combining solar heating and daytime radiative cooling through switching (including electrical regulation, thermal-responsive, and mechanical regulation) and collaborative modes. Subsequently, a few outlooks are listed below.

(1) Because long-spectral (from visible to mid-IR) transparent conductive electrodes are not readily available and the relationship between deposition structure and performance is unclear, it is still challenging to realize the efficient regulation of the solar and IR spectra simultaneously. Improving the regulatory range (or performance) is a bottleneck that urgently needs to be solved. In addition, electrical regulation often requires organic electrolytes, and their stability in the high-temperature heating state still requires further testing and design.
(2) VO_2_ and hydrogel materials are primarily used for thermal responsiveness. With VO_2_, it is difficult to control the solar energy spectrum because there is no noticeable color change during the VO_2_ phase transition. The challenge with hydrogels is that there is no way to regulate their IR emission. Innovative strategies, including materials, structures, and device designs, are required to address these issues.
(3) Reports of effective programmable meter-level devices for mechanical regulation demonstrate the potential of real-world applications. The durability of materials and devices and their application boundary conditions (such as economics, different climates, and building types) must be considered before applying them in real applications. Moreover, several techniques based on roll-to-roll and Janus methods merit additional investigation.
(4) The cooperative mode is still in the proof-of-concept stage. It is challenging to guarantee efficient heating and cooling performances simultaneously due to inefficient light and thermal management. Designing materials with high sunlight absorption and mid-IR transmission and designing better structures to dissociate the heating and cooling effects are promising strategies to resolve this issue. Additionally, innovative and collaborative modes are essential.
(5) For successful applications, the boundary conditions and energy-saving effects of dual modes must be carefully calculated for different climate environments. Examples include the effect of switching times on energy savings, the relationship between the energy consumption of switching and that of the maintaining state, and the energy consumption of buildings under various conditions.
